# State-dependent judgement bias in *Drosophila*: evidence for evolutionarily primitive affective processes

**DOI:** 10.1098/rsbl.2017.0779

**Published:** 2018-02-28

**Authors:** Amanda Deakin, Michael Mendl, William J. Browne, Elizabeth S. Paul, James J. L. Hodge

**Affiliations:** 1 Centre for Behavioural Biology, Bristol Veterinary School, University of Bristol, Langford, UK; 2 Centre for Multilevel Modelling, University of Bristol, Bristol, UK; 3 School of Physiology, Pharmacology and Neuroscience, University of Bristol, Bristol, UK

**Keywords:** affective states, judgement bias, *Drosophila*, fruit flies

## Abstract

Affective states influence decision-making under ambiguity in humans and other animals. Individuals in a negative state tend to interpret ambiguous cues more negatively than individuals in a positive state. We demonstrate that the fruit fly, *Drosophila melanogaster*, also exhibits state-dependent changes in cue interpretation. *Drosophila* were trained on a Go/Go task to approach a positive (P) odour associated with a sugar reward and actively avoid a negative (N) odour associated with shock. Trained flies were then either shaken to induce a purported negative state or left undisturbed (control), and given a choice between: air or P; air or N; air or ambiguous odour (1 : 1 blend of P : N). Shaken flies were significantly less likely to approach the ambiguous odour than control flies. This ‘judgement bias’ may be mediated by changes in neural activity that reflect evolutionarily primitive affective states. We cannot say whether such states are consciously experienced, but use of this model organism's versatile experimental tool kit may facilitate elucidation of their neural and genetic basis.

## 1. Introduction

Animal affective (emotional) states can be operationally defined as ‘states elicited by rewards and punishers' where rewards are stimuli that animals work to acquire and punishers are stimuli that they work to avoid [1]. This behaviourally grounded definition allows systematic study of animal affect despite lack of knowledge about whether such states, which we assume to be instantiated in neural activity, are consciously experienced.

Recently, there has been growing interest in the possibility that affective states, or their evolutionary precursors, exist in invertebrates [2–10]. For example, Anderson & Adolphs [4] identify what they call ‘emotion primitives’, general properties of affective states such as scalability, valence, persistence and generalization. Gibson *et al*. [5] argue that such characteristics can be observed in spontaneous responses of *Drosophila* to a repeated threatening visual cue. Likewise, the spontaneous behaviour of shocked crayfish in a variant of the elevated plus maze [3], or of *Drosophila* treated with diazepam in an open field test [9] appear similar to, respectively, ‘anxious’ or ‘relaxed’ behaviour shown by rodents in these tests.

Affective valence (positivity/negativity) is arguably the key defining characteristic of emotion. The ‘judgement bias’ (JB) test offers a way of measuring this that is generalizable across species [11,12]. Animals in positive affective states are predicted to show more positive judgements of ambiguous stimuli than those in negative states, a cognitive or judgement bias that is observed in humans [13] and may have adaptive value [14]. The JB test [15] has been used in many vertebrate species and, more recently, in social insects, with predicted judgement biases being observed in honeybees [2,10] and bumblebees [6]. Here we investigate whether such biases may also be observed in a non-hymenopteran insect, *Drosophila melanogaster*. If so, this would indicate that affect-related judgement biases may occur in insect species that lack a complex social organization, suggesting that this interplay between affect and decision-making is preserved across a wide phylogeny and hence, as hypothesised [14], is likely to have adaptive value. Moreover, it would open the way for studies of the neural basis of affective valence in this genetically tractable organism for which sophisticated tools including numerous inducible promoters, opto- and thermogenetics, and the potential for engineered mutations in every gene, are readily available.

We adapted well-established learning assays [16,17] to develop a JB test for *Drosophila*. Flies learnt to avoid an odour (negative; N) associated with shock and approach an odour (positive; P) associated with a sucrose reward. One group of flies were then shaken for 1 min while a second group were left undisturbed. Flies were tested to see whether they judged an ambiguous 1 : 1 blend of odours P and N positively (approach) or negatively (avoid). Shaking induces avoidance of associated colours in *Drosophila* [18,19]; therefore, it was predicted that shaking would induce a negative state resulting in a negative judgement bias as previously observed in honeybees [2,10].

## 2. Material and methods

### (a) Flies and apparatus

**Figure 1.**
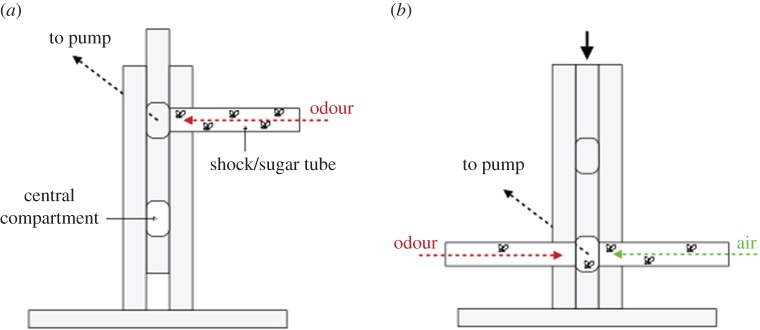
T-maze apparatus. A pump draws odours (red arrows) or air (green arrow) through the apparatus. (*a*) Training and (*b*) testing configuration for judgement bias assay (see text for details).

Subjects were 1–3-day-old white-eyed wild-type flies of the Canton-S-white strain. A well-established *Drosophila* T-maze classical conditioning apparatus [16,17] was used, consisting of two Plexiglas vertical columns containing a movable Plexiglas piece that housed a central compartment (lift) in which flies could be transferred between test tubes at upper and lower levels (figure 1). Flies were trained in the upper level tube to associate specific odours with either a positive (sucrose) or negative (shock) stimulus (figure 1*a*). Testing took place at the lower level (figure 1*b*), where flies were given a choice between two odours (initial testing), or an odour and air (judgement bias assay) presented simultaneously in two tubes to see which they approached. See electronic supplementary material for details.

### (b) Odours

We used odours that are widely employed in *Drosophila* studies: 4-methylcyclohexanol (MCH) and 3-octanol (OCT) diluted in mineral oil (Sigma-Aldrich) [16] at concentrations at which flies showed no preference for OCT versus MCH. After conducting initial aversive and appetitive learning tests, MCH was always paired with shock (negative odour; N) and OCT with sucrose (positive odour; P) for the judgement bias assay (details in the electronic supplementary information).

### (c) Judgement bias assay

After 90 s acclimatization in the upper tube, flies were trained by receiving MCH (N) paired with shock for 1 min, followed by 30 s in the lift, followed by OCT (P) paired with sucrose for 1 min. Flies were then moved to the lift for 30 s before being transferred into their original vials for 1 min. Of note, 50% of vials (shaken group; *n* = 15 vials) then experienced 1 min of shaking (Vortex-T Genie 2, Scientific Industries; 2800 r.p.m., approximately 1.17 m s^−1^, 1 s rest every 6 s) while the other 50% were not shaken (control; *n* = 15 vials). After a further 1 min in the vials, flies were transferred to the lower level of the apparatus and given a 120 s choice between: (a) air or P; (b) air or N; (c) air or a 1 : 1 blend of P and N (P : N). Each vial of flies completed one choice test only.

### (d) Statistical analysis

After testing, the number of flies in each tube was counted to determine whether they approached the odour presented (P, N, P : N) or air. The dependent variable was: (no. flies approaching odour/total no. of flies making a choice) × 100. Each vial was the unit of analysis. The proportion of flies tested that did not choose (remained in the lift) was recorded. Data were analysed using two-way ANOVA with main effects of odour, cue (P, N, P : N) and treatment (shaken, control) and a cue × treatment interaction. Post hoc tests consisted of simple main effects analysis with Bonferonni correction.

## 3. Results and discussion

As expected, *Drosophila* learnt to associate one odour with either shock or sucrose in the respective standard single-odour assays (electronic supplementary material, figure S1A,B). They also learnt to discriminate between one odour (MCH) associated with shock and another (OCT) associated with sucrose in a double-odour assay (electronic supplementary material, figure S1C). This allowed us to develop an active choice Go/Go judgement bias task in which flies had to choose to approach either an ambiguous odour (MCH : OCT mixture) or air, in contrast to Go/NoGo tasks previously used in insects [2,6,10]. Non-affect-related decreases in activity, or extinction of responses to cues, may favour NoGo responses in the latter tasks which can then be erroneously interpreted as a negative judgement. Go/Go tasks avoid this problem [20,21].

**Figure 2.**
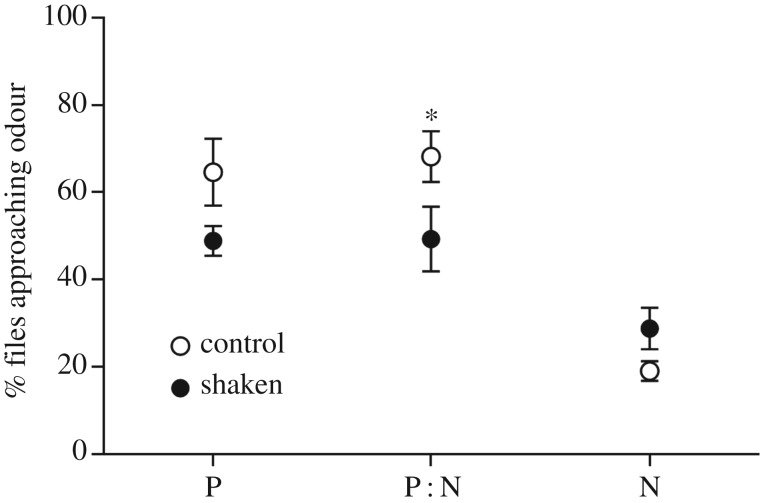
Judgement bias assay. Mean (±1 s.e.m.) percentage of control and shaken flies approaching P (sugar-associated), P : N (ambiguous blend) and N (shock-associated) odours compared with air. *p < 0.05.

Choice in the Go/Go task was influenced by a cue × treatment interaction (*F*_2,24_ = 3.93, *p* = 0.03; figure 2). A lower percentage of shaken flies approached the ambiguous P : N odour than control flies (mean difference ± s.e.m. = 18.92 ± 7.94, *F*_1,24_ = 5.68, *p* = 0.025), in support of our hypothesis. There was a non-significant trend for the same effect in P (sucrose-associated) odour tests (mean difference ± s.e.m. = 15.68 ± 7.94, *F*_1,24_=3.90, *p* = 0.060), but no difference for the N (shock-associated) odour (mean difference ± s.e.m. = 9.81 ± 7.94, *F*_1,24_ = 1.53, *p* = 0.228).

A significant effect of cue (*F*_2,24_ = 24.31, *p* < 0.001) reflected that flies were more likely to approach odour P than N (mean difference ± s.e.m. = 32.83 ± 5.61, *p* < 0.001) and hence that they discriminated between positive and negative cues. A lower percentage of flies approached odour N than ambiguous odour P : N (mean difference ± s.e.m. = 34.84 ± 5.61, *p* < 0.001), but approaches to odours P and P : N did not differ (mean difference ± s.e.m. = 2.01 ± 5.61, *p* = 1.00). This latter finding could indicate that (i) appetitive memory had not been formed or was disrupted, resulting in flies treating both P and P : N cues as ambiguous, or (ii) flies did associate odour P with rewarding sucrose, but perceived odour P : N (1 : 1 OCT/MCH blend) to be more similar to odour P (OCT) than to N (MCH). Explanation (i) appears unlikely because significantly more flies trained to associate odour P (OCT) with sucrose (control and shaken flies in the JB assay) chose OCT relative to air in comparison with naive untrained flies given a choice between OCT and air (odour preference data in the electronic supplementary material), suggesting that the former had indeed learnt the association (*t*-tests: control versus naive: *t*_7_ = 4.32, *p* = 0.003; shaken versus naive: *t*_7_ = 5.01, *p* = 0.002). Furthermore, the approximate velocity of shaken flies in this study (approx. 1.17 m s^−1^) was lower than that (2.1 m s^−1^) used to induce mild traumatic brain injury in *Drosophila* (a model of mild repetitive head injuries sustained during sport [22]) and any associated memory impairment. Moreover, flies shaken at similar velocities were able to associate shaking itself with colour discriminative cues, indicating that learning and memory mechanisms function effectively during this type of treatment [18,19]. Explanation (ii) thus appears more plausible (see [10] for a similar perceptual asymmetry), and future experiments would benefit from using a range of OCT : MCH blends to investigate which odour mixtures are treated as perceptually intermediate by flies.

The proportion of non-choosing flies was also affected by a cue × treatment interaction (*F*_2,24_ = 4.03, *p* = 0.03). A lower proportion of shaken flies made a choice in N tests (mean difference ± s.e.m. = 0.08 ± 0.018, *p* < 0.001) compared with non-shaken flies. A similar trend was seen in P tests (mean difference ± s.e.m. = 0.036 ± 0.018, *p* = 0.051), but there was no difference in P : N tests (mean difference ± s.e.m. = 0.009 ± 0.018, *p* = 0.602). Shaking may thus have increased uncertainty and decreased active choices in the trained conditions (P,N), but not when there was already inherent uncertainty (ambiguous P : N cue).

As in honeybees [2,10], short-term mechanical shaking induced a negative judgement of an ambiguous cue. Because a Go/Go task was used, this does not reflect an effect on activity levels but rather an alteration in the proportion of flies choosing to approach or avoid the ambiguous odour. This was also observed in response to the trained positive cue (cf. negative judgement of negative cue in honeybees [2]). The latter finding may indicate that, in addition to a lowered expectation of reward/increased expectation of punishment under ambiguity, shaken flies also showed a decreased *valuation* of reward predicted by the non-ambiguous positive cue. Further studies are needed to discriminate between these two possibilities (cf. [23]).

Our study provides the first evidence that a non-social insect, *Drosophila melanogaster*, shows judgement biases similar to those observed in Hymenoptera, and adds to data on spontaneous behaviour that may also indicate affective processes in this species [4,5,9]. We assume that these biases and behaviours are mediated by changes in molecular pathways and neural activity that may represent evolutionarily primitive affective states and are amenable to detailed genetic investigation in *Drosophila*, but we cannot say whether they are accompanied by conscious experience [7,24].

## Supplementary Material

Supplementary methods and results

## Data accessibility

Data available from Dryad Digital Repository: (http://dx.doi.org/10.5061/dryad.81r60) [25].

## References

[1] Rolls ET 2014 Emotion and decision-making explained. Oxford, UK: Oxford University Press.

[2] Bateson M, Desire S, Gartside SE, Wright GA 2011 Agitated honeybees exhibit pessimistic cognitive biases. Curr. Biol. 21, 1070–1073. (10.1016/j.cub.2011.05.017) 21636277 PMC3158593

[3] Fossat P, Bacqué-Cazenave J, De Deurwaerdère P, Delbecque J-P, Cattaert D 2014 Anxiety-like behaviour in crayfish is controlled by serotonin. Science 344, 1293–1297. (10.1126/science.1248811) 24926022

[4] Anderson DJ, Adolphs R 2014 A framework for studying emotions across species. Cell 157, 187–200. (10.1016/j.cell.2014.03.003) 24679535 PMC4098837

[5] Gibson WT et al. 2015 Behavioral responses to a repetitive visual threat stimulus express a persistent state of defensive arousal in *Drosophila*. Curr. Biol. 25, 1401–1415. (10.1016/j.cub.2015.03.058) 25981791 PMC4452410

[6] Perry CJ, Baciadonna L, Chittka L 2016 Unexpected rewards induce dopamine-dependent positive emotion-like state changes in bumblebees. Science 353, 1529–1532. (10.1126/science.aaf4454) 27708101

[7] Mendl M, Paul ES 2016 Bee happy. Science 353, 1499–1500. (10.1126/science.aai9375) 27708091

[8] Perry CJ, Baciadonna L 2017 Studying emotion in invertebrates: what has been done, what can be measured and what they can provide. J. Exp. Biol. 220, 3856–3868. (10.1242/jeb.151308) 29093185

[9] Mohammed F, Aryal S, Ho J, Stewart JC, Norman NA, Tan TL, Eisaka A, Claridge-Chang A 2016 Ancient anxiety pathways influence *Drosophila* defense behaviors. Curr. Biol. 26, 981–986. (10.1016/j.cub.2016.02.031) 27020741 PMC4826436

[10] Schluns H, Welling H, Federici JR, Lewejohann L 2016 The glass is not yet half empty: agitation but not *Varroa* treatment causes cognitive bias in honey bees. Anim. Cogn. 20, 233–241. (10.1007/s10071-016-1042-x) 27699501

[11] Mendl M, Burman OHP, Parker RMA, Paul ES 2009 Cognitive bias as an indicator of animal emotion and welfare: emerging evidence and underlying mechanisms. Appl. Anim. Behav. Sci. 118, 161–181. (10.1016/j.applanim.2009.02.023)

[12] Bethell EJ 2015 A ‘how-to’ guide for designing judgment bias studies to assess captive animal welfare. J. Appl. Anim. Welf. Sci. 18, S18–S42. (10.1080/10888705.2015.1075833) 26440495

[13] Paul ES, Harding EJ, Mendl M 2005 Measuring emotional processes in animals: the utility of a cognitive approach. Neurosci. Biobehav. Rev. 29, 469–491. (10.1016/j.neubiorev.2005.01.002) 15820551

[14] Mendl M, Burman OHP, Paul ES 2010 An integrative and functional framework for the study of animal emotion and mood. Proc. R. Soc. B 277, 2895–2904. (10.1098/rspb.2010.0303) PMC2982018 20685706

[15] Harding EJ, Paul ES, Mendl M 2004 Cognitive bias and affective state. Nature 427, 312 (10.1038/427312a) 14737158

[16] Tully T, Quinn WG 1985 Classical conditioning and retention in normal and mutant *Drosophila*. J. Comp. Physiol. A 157, 263–277. (10.1007/BF01350033) 3939242

[17] Schwaerzel M, Monastirioti M, Scholz H, Friggi-Grelin F, Birman S, Heisenberg M 2003 Dopamine and octopamine differentiate between aversive and appetitive olfactory memories in *Drosophila*. J. Neurosci. 23, 10 495–10 502. 10.1523/JNEUROSCI.23-33-10495.2003 PMC6740930 14627633

[18] Menne D, Spatz HC 1977 Color vision in *Drosophila melanogaster*. J. Comp. Physiol. 114, 301–312. (10.1007/BF00657325)

[19] Bicker G, Reichert H 1978 Visual learning in a photoreceptor degeneration mutant of *Drosophila melanogaster*. J. Comp. Physiol. 127, 29–38. (10.1007/BF00611923)

[20] Matheson SM, Asher L, Bateson M 2008 Larger, enriched cages are associated with ‘optimistic’ response biases in captive European starlings (*Sturnus vulgaris*). Appl. Anim. Behav. Sci. 109, 374–383. (10.1016/j.applanim.2007.03.007)

[21] Jones SM, Paul ES, Dayan P, Robinson ESJ, Mendl M 2017 Pavlovian influences on learning differ between rats and mice in a counter-balanced Go/NoGo judgement bias task. Behav. Brain Res. 331, 214–224. (10.1016/j.bbr.2017.05.044) 28549647 PMC5480777

[22] Barekat A et al. 2016 Using *Drosophila* as an integrated model to study mild repetitive traumatic brain injury. Sci. Rep. 6, 25252 (10.1038/srep25252) 27143646 PMC4855207

[23] Iigaya K, Jolivald A, Jitkrittum W, Gilchrist ID, Dayan P, Paul E, Mendl M 2016 Cognitive bias in ambiguity judgements: using computational models to dissect the effects of mild mood manipulation in humans. PLoS ONE 11, e0165840 (10.1371/journal.pone.0165840) 27829041 PMC5102472

[24] Barron AB, Klein C 2016 What insects can tell us about the origins of consciousness. Proc. Natl Acad. Sci. USA 113, 4900–4908. (10.1073/pnas.1520084113) 27091981 PMC4983823

[25] Deakin A, Mendl M, Browne WJ, Paul ES, Hodge JJL 2018 Data from: State-dependent judgement bias in *Drosophila*: evidence for evolutionarily primitive affective processes *Dryad Digital Repository*. (10.5061/dryad.81r60) PMC5830672 29491031

