# Supplementary material for: State-dependent judgement bias in Drosophila: evidence for evolutionarily primitive affective processes
Source: Biol Lett. 2018 Feb 28;14(2):20170779. doi: 10.1098/rsbl.2017.0779 (PMC5830672; doi:10.1098/rsbl.2017.0779)
Supplement: Supplementary methods and results [file rsbl20170779supp1.docx]

Supplementary material for:

State-dependent judgement bias in *Drosophila*: evidence for evolutionarily primitive affective processes

Amanda Deakin, Michael Mendl, William J. Browne, Elizabeth S. Paul & James J.L. Hodge

**Methods**

1. **Fly husbandry**

The flies were kept in transparent plastic bottles (250 ml) containing a standardised quantity of fly food consisting of maize, dried yeast, soya flour, malt extract, molasses, agar, propionic acid and P-hydroxybenzoate (an anti-fungal). Flies were kept at a constant temperature of 25°C on a 12hr:12hr light:dark cycle. Before behaviour experiments, flies were transferred to smaller plastic vials (2.5cm x 9.5cm) containing 4ml of 1% agar and a standardised amount of anti-fungal in which they were starved for 18-20 hours (mean number of flies per tube = 57, range = 24 – 85). Starved flies were required for appetitive conditioning as the memory performances of *Drosophila* in appetitive learning memory tasks are directly linked to the degree of motivation to find food [S1]. Therefore, all flies were starved on agar for use in the experiments reported here to control for any unwanted effects of starvation. During this time, flies were allowed to acclimatise to the conditions under which behaviour experiments were conducted (25°C, 70% relative humidity).

1. **Test apparatus**

Olfactory learning was tested using a well-established *Drosophila* classical conditioning paradigm in a T-maze [S2-3]. The T-maze consisted of two Plexiglas vertical columns containing a movable Plexiglas piece that housed a lift in which flies could be transferred between upper and lower levels. Flies were trained in the upper level (Figure 1A). Shocks were administered in a tube containing an electrifiable grid attached to a generator (60V shock consisting of 1.25s pulses with 3.25s inter-pulse intervals). Sucrose was administered in a tube containing a 4.5cm x 8.5cm piece of filter paper that had been saturated previously with 2M sucrose (in 400µl distilled water), left to dry and re-humidified with 400µl distilled water before insertion into the tube. A control tube was treated with distilled water only. In both cases, training tubes were modified test tubes attached to an air supply from which odours could be drawn over the flies. Testing took place in the lower level of the apparatus (Figure 1B) where flies were given a choice between two odours (initial testing) or odour and air (judgement bias assay) presented simultaneously in two tubes. All experiments took place under dim red light allowing the experimenter but not the flies to see, to minimise the effects of phototaxis and help the flies concentrate on olfactory inputs. The times given for acclimatisation, odour presentation, and odour choice were based on published work [S2-3].

1. **Odour balancing**

***Odour bias***

Flies were tested for innate odour avoidance bias using a simple choice assay. In this assay flies were acclimatised to conditions in the lift for 90 seconds and then were given a choice between MCH in one tube and OCT in the other tube at the lower level of the T-maze apparatus. The choice period was 120s after which flies were trapped in their chosen tubes and counted. The percentage of flies that avoided each odour was calculated. If no innate bias was present, the flies should be distributed with roughly 50% in each odour tube.

***Odour preference***

Flies’ preference for the two odours compared to fresh air pulled through plain mineral oil was tested. In this assay, flies were acclimatised to conditions in the lift for 90 seconds and then given a choice between either MCH or OCT in one tube vs fresh air in the other tube at the lower level of the T-maze apparatus. The choice period was 120s after which flies were trapped in their chosen vials and counted. The percentage of flies that avoided the odour (approached air) was calculated.

1. **Initial testing**

***Aversive learning***

A standard protocol for aversive learning was followed [S2]. After a 90s acclimatisation period with no odour or shocks in the upper tube, flies were trained by receiving one odour (negative; N) with shocks (60V shock consisting of 12 x 1.25s pulses with 3.25s inter-pulse intervals) for 1 minute followed by a 30s rest then a second odour (positive; P) with no shocks for 1 minute. For half of the vials tested, MCH was paired with shocks and OCT with no shocks and for the other half of the vials the opposite was true. Flies were rested for a further 30s before being transferred into their original vials. Flies were rested for a retention period of 1 hour between training and testing. During testing, flies were transferred into the lift for a 90s acclimatisation period before being moved to the lower portion of the T-maze and given a 120s choice between the two odours, each presented in separate tubes (with no accompanying reinforcers). After 120s, flies were trapped in their chosen tubes and the number of flies choosing the P odour compared to the N odour was counted.

***Appetitive learning***

Appetitive learning methods were adapted from [S3]. Shortly before training, the sucrose and distilled water (control) filter papers were humidified using 400µl of distilled water and inserted into a test tube for use in the T-maze. Flies were transferred to the upper level of the apparatus and first given a 90s acclimatisation period in a tube with no odour or sucrose present. Flies were then transferred to the lift and the tube containing sucrose filter paper was inserted into the apparatus. Flies were moved to this tube to receive 1-minute exposure to one odour (Positive; P) with sucrose paper followed by a 30s rest in the lift. A tube containing distilled water filter paper was then inserted into the apparatus and flies were exposed to this for 1-minute with a second odour (Negative; N). For half of the vials tested, MCH was paired with sucrose and OCT with no sucrose and for the other half of the vials the opposite was true. Flies were rested for a further 30s before being transferred into their original vials. Flies were left for a retention period of 1h between training and testing. During testing, flies were transferred into the lift for a 90s acclimatisation period and then moved to the lower portion of the T-maze where they were given a 120s choice between the two odours, each in separate tubes (with no sucrose). After 120s, flies were trapped in their chosen tubes and the number of flies choosing the P compared to the N odour were counted.

***Combining aversive and appetitive learning***

In the combined assay, aversive and appetitive learning were combined into one assay where shock was predicted by MCH (negative; N) and sucrose was predicted by OCT (positive; P). The same protocols as above were used. After preliminary testing, N presentations always preceded P presentations to avoid any postprandial effects. After training on the combined assay, flies were given a 120s choice between the P and N odours, each in different tubes, and then trapped in their tubes and counted.

1. **Statistical analysis for odour balancing and initial testing experiments**

Data were obtained after tests by counting the number of flies in each tube under CO_2_ anaesthesia using a dissecting microscope. Flies that avoided the shock-associated odour and approached the sucrose-associated one were deemed ‘correct’ whilst those that did the opposite were deemed ‘incorrect’. Each vial was the unit of analysis and provided one value for the percentage of flies out of those that made a choice whose choice was ‘correct’. Statistical analysis was carried out in SPSS statistics version 24. Data were checked for normality and homogeneity of variance. One-sample t-tests were used to check whether the percentage of flies approaching each odour was significantly different from 50% in odour bias tests and to test whether learning had occurred (whether significantly more than 50% flies made the ‘correct’ decision; e.g. approached the odour paired with sucrose) in aversive and appetitive learning and combined learning assays. An independent t-test was used to check whether the preference for each odour compared to air was significantly different. A one-way ANOVA was used to compare mean scores from aversive conditioning, appetitive conditioning and combined conditioning.

**Results**

1. **Odour balancing**

***Odour bias***

Odours were found to be balanced (fly distribution was not significantly different from 50%) at a concentration of 80:10000 (mean OCT approach ± SEM = 45.68 ± 3.42; mean MCH approach ± SEM = 54.32 ± 3.42; One sample t test: t_7_ = 1.26, P = 0.25; n_OCT_ = 8; n_MCH_ = 8). These concentrations were used in further assays.

***Odour preference***

Both odours (OCT 80:10000, MCH 80:10000) were avoided by a similar percentage of flies when given the choice between the odour and air (mean OCT avoidance ± SEM = 74.89 ± 2.99; mean MCH avoidance ± SEM = 64.63 ± 5.90; Independent t-test: t_6_= 1.55, P = 0.17; n_OCT_ = 4; n_MCH_ = 4).

1. **Initial testing**

***Aversive conditioning***

In aversive conditioning, OCT and MCH were alternately used as the shock-associated odour and neutral odour. Both conditions led to effective learning (One sample t-test - OCT: t_6_ = 7.21, P < 0.01; MCH: t_6_ = 6.30, P < 0.01; Figure S1A).

***Appetitive conditioning***

Learning occurred when OCT was paired with sucrose (One sample t-test: t_3_ = 7.27, P < 0.01). When MCH was paired with sucrose, the percentage of flies approaching the positive odour was also significantly different from zero but in an unexpected direction (flies approached OCT) (One sample t-test: t_3_ = 7.86, P < 0.01; Figure S1B). Consequently, it was decided that for the combined aversive and appetitive learning assay, sucrose would be paired with OCT and shock would be paired with MCH. As these odours were balanced with respect to naïve preference, differences in preference after conditioning should be due to learning.

***Combining aversive and appetitive conditioning***

In the combined assay, flies successfully learnt that OCT predicted sucrose whereas MCH predicted a shock (One sample t-test: t_3_= 8.95, P < 0.01). This combined learning assay did not yield significantly different results compared to singular aversive or appetitive learning (One way ANOVA: F_2, 12_ = 0.24 P = 0.79; Figure S1C).

**References**

S1. Krashes MJ, DasGupta S, Vreede A, White B, Armstrong JD, Waddell S. 2009 A Neural Circuit Mechanism Integrating Motivational State with Memory Expression in *Drosophila*. *Cell* **139**, 416–427. (doi:10.1016/j.cell.2009.08.035)

S2. Tully T, Quinn WG. 1985 Classical conditioning and retention in normal and mutant *Drosophila*. *Journal of Comparative Physiology A* **157**, 263–277.

S3. Schwaerzel M, Monastirioti M, Scholz H, Friggi-Grelin F, Birman S, Heisenberg M. 2003 Dopamine and octopamine differentiate between aversive and appetitive olfactory memories in *Drosophila*. *The Journal of Neuroscience* **23**, 10495–10502.


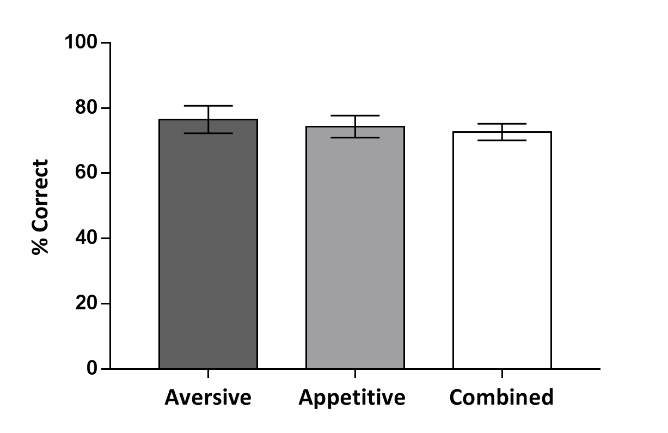

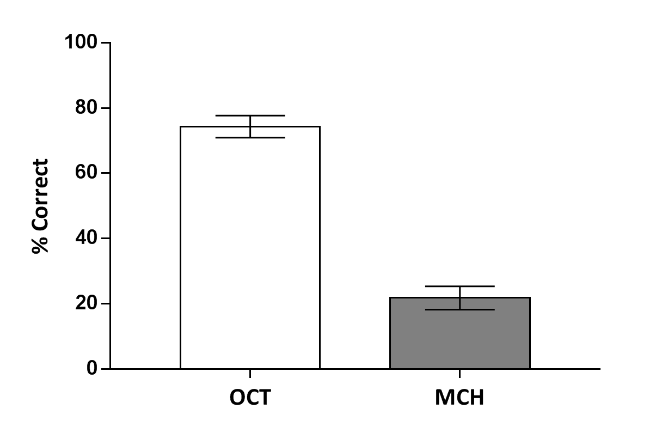

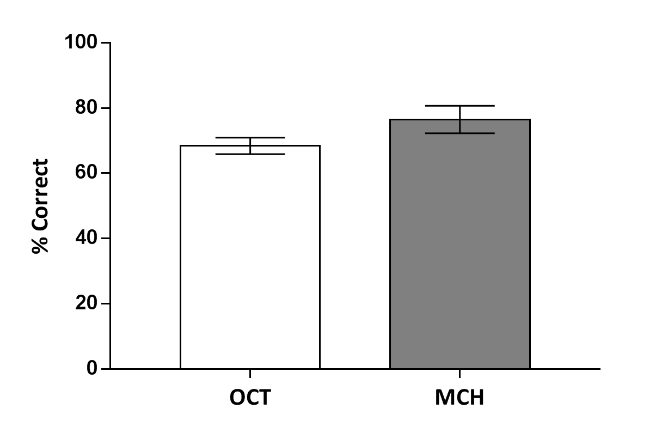


**A**

**B**

**C**

**Figure S1. Mean (± SEM) percentage of choosing flies that made the ‘correct’ choice in aversive conditioning, appetitive conditioning and combined conditioning assays.** **(A)** Aversive conditioning - percentage of flies making the correct choice (avoiding odour) when OCT was associated with shock compared to when MCH was used as the shock-associated odour; n_OCT_ = 7, n_MCH_ = 7. **(B)** Appetitive conditioning – percentage of flies making the correct choice (approaching odour) when OCT was associated with sucrose compared to when MCH was used as the sucrose-associated odour; n_OCT_ = 4, n_MCH_ = 4. **(C)** Combined conditioning – percentage of flies making the correct choice after singular aversive (MCH paired with shock) or appetitive (OCT paired with sucrose) learning compared with combined learning (OCT paired with sucrose *and* MCH paired with shock); n_aversive_ = 7, n_appetitive_ = 4, n_combined_ = 4.
